# Pulsatile flow increases METTL14-induced m^6^A modification and attenuates septic cardiomyopathy: an experimental study

**DOI:** 10.1097/JS9.0000000000001402

**Published:** 2024-03-27

**Authors:** Shenyu Zhu, Kai Wang, Zhexuan Yu, Wei Tang, Yu Zhang, Shafiu A. Shinge, Yongjia Qiang, Hangyu Liu, Jianfeng Zeng, Kun Qiao, Chi Liu, Guanhua Li

**Affiliations:** a Department of Thoracic Surgery, The First Affiliated Hospital of Gannan Medical University, Ganzhou; b Zhejiang Chinese Medical University, Hangzhou; c Department of Pathology, Guangdong Provincial Hospital of Chinese Medicine, The Second Affiliated Hospital of Guangzhou University of Chinese Medicine; d Integrated Hospital of Traditional Chinese Medicine of Southern Medical University; e Department of Cardiovascular Surgery, Guangdong Cardiovascular Institute, Guangdong Provincial People’s Hospital, Guangdong Academy of Medical Sciences; f Department of Cardiovascular Surgery, Sun Yat-sen Memorial Hospital, Sun Yat-sen University; g Department of Anesthesiology, Sun Yat-sen Memorial Hospital, Sun Yat-sen University, Guangzhou, Guangdong; h Department of Thoracic Surgery, The Third People’s Hospital of Shenzhen; i Department of Cardiovascular Surgery, the 8th Affiliated Hospital of Sun Yat-sen University, Shenzhen; j Department of Nephrology, Sichuan Academy of Medical Science and Sichuan Provincial People’s Hospital, Sichuan Renal Disease Clinical Research Center, University of Electronic Science and Technology of China, Chengdu, People’s Republic of China

**Keywords:** extracorporeal membrane oxygenation, microcirculation, N^6^-methyladenosine, pulsatile flow, septic cardiomyopathy

## Abstract

**Introduction:**

Septic cardiomyopathy is a sepsis-mediated cardiovascular complication with severe microcirculatory malperfusion. Emerging evidence has highlighted the protective effects of pulsatile flow in case of microcirculatory disturbance, yet the underlying mechanisms are still elusive. The objective of this study was to investigate the mechanisms of N^6^-methyladenosine (m^6^A) modification in the alleviation of septic cardiomyopathy associated with extracorporeal membrane oxygenation (ECMO)-generated pulsatile flow.

**Methods:**

Rat model with septic cardiomyopathy was established and was supported under ECMO either with pulsatile or non-pulsatile flow. Peripheral perfusion index (PPI) and cardiac function parameters were measured using ultrasonography. Dot blot assay was applied to examine the m^6^A level, while qRT-PCR, Western blot, immunofluorescence, and immunohistochemistry were used to measure the expressions of related genes. RNA immunoprecipitation assay was performed to validate the interaction between molecules.

**Results:**

The ECMO-generated pulsatile flow significantly elevates microcirculatory PPI, improves myocardial function, protects the endothelium, and prolongs survival in rat models with septic cardiomyopathy. The pulsatile flow mediates the METTL14-mediated m^6^A modification to zonula occludens-1 (ZO-1) mRNA (messenger RNA), which stabilizes the ZO-1 mRNA depending on the presence of YTHDF2. The pulsatile flow suppresses the PI3K–Akt signaling pathway, of which the downstream molecule Foxo1, a negative transcription factor of METTL14, binds to the METTL14 promoter and inhibits the METTL14-induced m^6^A modification.

**Conclusion:**

The ECMO-generated pulsatile flow increases METTL14-induced m^6^A modification in ZO-1 and attenuates the progression of septic cardiomyopathy, suggesting that pulsatility might be a new therapeutic strategy in septic cardiomyopathy by alleviating microcirculatory disturbance.

## Introduction

Highlights

- The ECMO (extracorporeal membrane oxygenation)-generated pulsatility attenuates septic cardiomyopathy.
- Pulsatile flow triggers METTL14-induced m^6^A modification in ZO-1.
- Pulsatility sustains m^6^A modification by suppressing PI3K–Akt signaling pathway.
- Foxo1 binds to the METTL14 promoter and inhibits m^6^A modification.
- Pulsatility is a new strategy for alleviating microcirculatory disturbance.

Septic cardiomyopathy, characterized by ventricular dysfunction induced by sepsis, is a critical cardiovascular complication with poor clinical outcomes. The pathophysiological alterations of septic cardiomyopathy are complicated, including microvascular disorder, impaired myocardial contractability, inflammatory cascade, and myocardial metabolic disturbance^1^. Treatment options for this lethal complication are limited, suggesting the importance of adopting new therapeutic approaches. Microcirculation is the main determinant and ideal endpoint for resuscitation, while its malperfusion is associated with increased mortality and morbidity for sepsis^2^. During septic cardiomyopathy, microcirculatory malperfusion contributes to endothelial dysfunction and coagulation system activation, with amplification of immune responses ultimately leading to organ damage, microcirculatory failure, and death^3,4^. Therefore, strategies avoiding microcirculatory malperfusion may benefit patients with septic cardiomyopathy.

Mounting evidence has highlighted the positive effects of pulsatile flow when microcirculation is disturbed during cardiopulmonary bypass, extracorporeal membrane oxygenation (ECMO), and cardiogenic shock^5–7^. Pulsatile flow enhances the microcirculatory perfusion by providing energy equivalent pressure and surplus hemodynamic energy, while biologically it alleviates the production of proinflammatory cytokines^8^. Previously study revealed that pulsatile flow up-regulates the expression of zonula occludens-1 (ZO-1) and protects the endothelial integrity^7^.

Studies of pulsatile flow applied to the setting of septic cardiomyopathy are rare. Kim *et al*.^9^ investigated possible effects of pulsatility in septic mice using body vibration and pulsatile flow reduced the production of interleukin-1β and tumor necrosis factor-α. Similarly, pulsatile flow induced by body periodic acceleration preserved enzyme nitric oxide synthase (eNOS) levels and reduced proinflammatory cytokines^10^. It is noteworthy that pulsatile flow was theorized to be possible treatment adjuncts against COVID-19^11,12^. However, the interrupted and unsteady nature of the abovementioned exercise-induced pulsatile flow limits the potentiality of future application in septic cardiomyopathy. Recent studies showed that pulsatile flow provided by ECMO steadily and successfully supports microcirculation and suppresses endothelial activation in animal models^7,8^. Against this background, it is necessary to explore if pulsatile flow has therapeutic effects on septic cardiomyopathy.

N^6^-methyladenosine (m^6^A), one of the most common modifications in mRNAs (messenger RNA) and non-coding RNAs, regulates RNA splicing, translation, and stability^13^. Emerging studies reported that m^6^A modification participates in various pathophysiological processes, including inflammation and sepsis^14^. Shen *et al*.^15^ revealed that downregulation of METTL3 and decreased m^6^A levels in lncRNA-XR_343955 activate inflammatory responses and interfere with endothelial integrity and function during sepsis. Zhang *et al*.^16^ found that YTHDF1, a known m^6^A reader, promotes the NLRP3 ubiquitination and suppresses inflammation in mouse models with septic shock. Whether pulsatile flow influences the m^6^A modification of mRNA and its underlying mechanisms remains obscure.

In the current study, we used animal models and cardiac microvascular endothelial cells (CMVECs) to simulate septic cardiomyopathy, with pulsatile flow provided by an ECMO circuit and a bioreactor. The role of m^6^A modification in this pathophysiological process was thoroughly investigated. The aim of this experimental study was to evaluate whether pulsatility attenuates septic cardiomyopathy and investigate detailed mechanisms.

## Materials and methods

### Animals

The Sprague–Dawley (SD) rats (10 weeks old, ~200 g) were purchased and cared for under specific pathogen-free (SPF) conditions. The experimental protocol was approved by the Institutional Animal Care Committee of our institution (reference no. KY-Z-2022-2553-01) in compliance with the ARRIVE, Supplemental Digital Content 1, http://links.lww.com/JS9/C240 (Animals in Research: Reporting In Vivo Experiments) guideline^17^. The septic cardiomyopathy rat model was created with 20 mg/kg of *Escherichia coli* lipopolysaccharide (Sigma, St. Louis, MO, USA) as suggested elsewhere^18^. The rats were then monitored by echocardiography until the left ventricular ejection fraction (LVEF) was reduced to 30% of the baseline level.

### Anesthesia, surgical procedure, and the ECMO circuit

Sixteen rat models with septic cardiomyopathy were randomly allocated into two groups: the non-pulsatile group and the pulsatile group. The arterial line and venous line were established surgically using the tail artery and tail vein, respectively. The rats were then anesthetized with 1% pentobarbital sodium (50 mg/kg) intraperitoneal induction and inhalation of 5.0% isoflurane mixed air, followed by orotracheal intubation with a 16-G catheter, which was connected to the Reward R407 animal ventilator (Reward Life Science & Technology Co., Ltd., Shenzhen, China), with volume-controlled ventilation (frequency: 70 times/min, tidal volume 10 ml/kg). The right common artery was cannulated with a 22G catheter (Terumo, Eschborn, Germany) and served as the ECMO inflow. A 18 G venous cannula (Togo Medikit Co., Ltd., Tokyo, Japan) was then inserted via the right internal jugular vein, advanced into the right atrium, and used as the venous return for the ECMO circuit. The ECMO circuit was primed with 8 ml Ringer’s solution and 500 IU of heparin. The circuit was established by a peristaltic pump with a flow damper (PreFluid, Changzhou, Jiangsu, China) providing pulsatile or non-pulsatile flow as described previously^7^, an animal membrane oxygenator (Xijing Medical Instrument, Xi’an, China), and the circuit tubing line (Xijing Medical Instrument, Xi’an, China). The ECMO circuit was maintained for 6 h.

### Ultrasonographic assessments

We used the peripheral perfusion index (PPI) to measure tissue perfusion in animals with septic cardiomyopathy. PPI is the ratio between the microcirculatory pulsatile component and the non-pulsatile component of the tissue when the light goes through the pulse oximetry^19^. PPI was assessed using a laser perfusion monitor (PeriFlux System 5000, Perimed, Stockholm, Sweden) attached to the peripheral tissue of the animals. The parameters were obtained at a frequency of 32 Hz and were measured by an analyzing software (PeriSoft for Windows, Perimed, Stockholm, Sweden), as described previously^19,20^.

Echocardiography was done by an experienced examiner blinded to group allocation. The LVEF and fraction of short-axis shortening (FS) were measured in M-mode with an ultrasound system (GE Healthcare, Atlanta, GA, USA). Parameters were measured thrice and averaged.

### Pathological analyses

Heart tissues obtained from the experimental animals were fixed in 10% buffered formalin, embedded in paraffin, and cut into sections, which were stained with hematoxylin and eosin (H&E). For immunohistochemistry (IHC) analysis, the heart tissues were extracted and fixed with 4% paraformaldehyde, with incubation of primary antibodies against ZO-1 (29274; Signalway Antibody LLC, Greenbelt, MD, USA) and METTL14 (ab223090; Abcam, Cambridge, MA, USA). Images were detected with a Zeiss Axio Observer (Zeiss, Oberkochen, Germany). Masson staining was performed according to protocols of the Masson’s Trichrome Staining Kit (abs9348; Absin Bioscience Inc., Shanghai, China).

### Cell culture and in vitro pulsatile experiments

Rat cardiac microvascular endothelial cells (CMECs) were isolated from the left ventricle of the euthanized SD rats. Briefly, the pieced left ventricle was washed in phosphate-buffered saline (PBS) and digested in 0.1% trypsin (PB180225; Procell, Wuhan, China). The cells were centrifuged at 300*g* for 10 min and were incubated in the CMEC medium (CM-R135; Procell, Wuhan, China) with 10% fetal bovine serum (10099141C; Gibco, Rockville, MD, USA) at room temperature. Cell mediums were replaced every 3 days.

As described previously^7^, we used the Flexcell apparatus (Flexcell Inc., McKeesport, PA, USA) to generate pulsatility to CMECs. Seeded CMECs (1×10^5^ cells/well) on Flexcell plates were treated with continuous flow (0 dyne/cm^2^) or pulsatile flow (3 dyne/cm^2^) for 6 h, with frequency and flow rate of 1 Hz and 2 mL/min, respectively.

### m^6^A dot blot

CMECs were exposed to pulsatile flow or non-pulsatile flow for the experimental settings. Total RNAs of CMECs were isolated using the TRIzol reagent (15596018; Invitrogen, Carlsbad, CA, USA). The poly (A)^+^ mRNAs were accrued according to the protocols of the magnetic messenger RNA isolated kit (S1550S; New England Biolabs, Ipswich, MA, USA). Using the specific m^6^A antibody (202003; Synaptic Systems, Goettingen, Germany), the mRNAs spotted on the Hybond N^+^ membranes were detected by dot blot assays.

### Plasmid reconstruction and lentivirus infection

The METTL14 promoter region (sequence −2000 bp to 100 bp) was constructed to the pGL3-basic vector (E1751; Promega, Beijing, China), in which the reporter plasmid was established. To construct shRNA (short hairpin RNA) of Foxo1, obligos for Foxo1 mRNA (Supplementary Table 2, Supplemental Digital Content 2, http://links.lww.com/JS9/C241) were synthesized (Genepharma, Soochow, China) and cloned into the pLKO.1-puro vector (QP1765; Qiyunbio. Inc., Shenzhen, China). The full-length OFR of rat METTL14 (NM_001399249.1) and Foxo1 (NM_001191846.3) were cloned into the pcDNA3.1 (+) vector (QP1673; Qiyunbio. Inc., Shenzhen, China) and pcDNA3.1 (+)/myc-His C vector (LM-1972; LMAI Bio Corp., Shanghai, China), respectively. To dephosphorylate Foxo1, Foxo1 gene S250A and S313A point mutation plasmids: pcDNA3.1 (+)/myc-Foxo1(S250A) and pcDNA3.1 (+)/myc-Foxo1(S313A) were constructed and verified by DNA sequencing. Centrifuged at 4000*g* for half an hour, the resuspended lentivirus was applied for target cell infection, after which cells were administered with puromycin (P8230; Solarbio Life Sciences Co., Ltd., Beijing, China) for 5 days.

### Quantitative reverse transcription-polymerase chain reaction (qRT-PCR)

After the extraction of total RNA by TRIzol reagent, reverse transcription was done using the Superscript III reverse transcriptase (18080-044; Invitrogen, Carlsbad, CA, USA). PCR samples consisted of complementary DNAs, power-SYBR Green Mix (4368706; Applied Biosystems, Warrington, UK), and specific primers (Supplementary Table 1, Supplemental Digital Content 2, http://links.lww.com/JS9/C241). Using the LightCycler 480 (Roche, Basel, Switzerland), qRT-PCR was done thrice for each experiment. The expression levels were normalized to GADPH (glyceraldehyde 3-phosphate dehydrogenase) as the internal control.

### Luciferase reporter assay

The CMECs were transfected with pcDNA3.1 (+)/myc-His C vector (LM-1972; LMAI Bio Corp., Shanghai, China) control or pcDNA3.1 (+)/myc-His C-Foxo1 plasmid, followed by co-transfection reporter plasmids including pGL3-basic-METTL14 promoter vector and TK-renilla vector. One day after transfection, the luciferase reporter assays were performed according to the protocol of Dual-Luciferase Reporter System (E1960; Promega, Beijing, China). Normalizing Firefly luciferase activity to that of Renilla signals, the transfection efficiency was calculated. Data for luciferase reporter assays were independently measured thrice.

### RNA immunoprecipitation (RIP)

RIP was done using the EZ-Magna RIP kit (17-701; Merck Millipore, Burlington, MA, USA). Cells were lysed in the RIP lysis buffer and were then centrifuged at 12 000*g* at 4°C for 30 min. The supernatant was incubated with METTL14 or YTHDF2 (ab246514; Abcam, Cambridge, MA, USA) antibodies or the IgG antibody (control) that protein A/G conjugated magnetic beads (HY-K0202; MedChemExpress, Shanghai, China) at 4°C overnight. Immunoprecipitation (IP) was performed with beads for bound RNAs. Beads were washed with RIP buffer four times and were then treated with 10% SDS, Proteinase K (HY-108717; MedChemExpress, Shanghai, China), and RIP buffer at 55°C for half an hour. RNAs in the Input group or IP were recovered with TRIzol reagent and were subject to qRT-PCR.

### Western blot

RIPA lysis buffer (HY-K1001; MedChemExpress, Shanghai, China) was applied for protein extraction. The total proteins were subject to the 10% sodium dodecyl sulfate-polyacrylamide gel electrophoresis, followed by transfer onto a polyvinylidene difluoride membrane, which was immersed in 5% fat-free milk. Membranes were subsequently treated at 4°C overnight with the indicated primary antibodies. Secondary antibodies were subsequently incubated for 1 h at room temperature. Finally, protein detection was performed with the ECL reagent (RPN2108; Amersham Life Science, Piscataway, NJ, USA). Blots were quantitatively analyzed with the Image J software (NIH, MD, USA). All experiments were done thrice.

### Immunofluorescence

For tissue immunofluorescence, the heart tissues sections were treated with anti-CD31 (Sanyou Bio, Shanghai, China) and anti-ZO-1 (29274; Signalway Antibody LLC, Greenbelt, MD, USA) at 4°C overnight, the secondary antibody (ab6721; Abcam, Cambridge, MA, USA) was then added, followed by 4′,6-diamidino2-phenylindole (DAPI) (ab228549; Abcam, Cambridge, MA, USA) staining. For cellular immunofluorescence, CMECs were fixed with 4% paraformaldehyde for 30 min at room temperature, followed by incubation of 0.2% Triton X-100 (HY-Y1883A; MedChemExpress, Shanghai, China) containing 3% bovine serum albumin for 1 h. Samples were incubated with primary antibodies against ZO-1 and CD31 at room temperature overnight. The slides were finally incubated with secondary antibodies and DAPI. Immunofluorescent images were obtained and photographed with a fluorescence microscope (BX51; Olympus, Tokyo, Japan).

### Chromatin immunoprecipitation (ChIP) assay

After 48 h of transfection with the control vector or pcDNA3.1 (+)/myc-His C-Foxo1 vector in CMECs, the ChIP assay was conducted using the ChIP kit (P2080S; Beyotime, Shanghai, China). CMECs were washed with PBS and were cultivated for 30 min with formaldehyde added. With the addition of glycine, the CMECs were washed and centrifuged at 300*g* at 4°C. The cells were then resuspended in 1 ml sonication buffer. After 15 min centrifugation at 15 000*g* at 4°C, we collected the supernatant, of which 10% was used as input, and 90% was treated with anti-MYC-tag antibody (EM1902-38; Huabio, Hangzhou, China) at 4°C overnight. With the Protein A/G Magnetic Beads added, immunoprecipitated DNAs were extracted with the PCR purification kit (28106; Qiagen, Dusseldorf, Germany) and were examined by qRT-PCR.

### Assays for mRNA stability

CMECs exposed to pulsatile flow or non-pulsatile flow were treated with 10 μg/ml of actinomycin D (HY-17559; MedChemExpress, Shanghai, China) to suppress RNA formation. CMECs were harvested and total RNAs were extracted as mentioned above at different time points. The remaining levels of ZO-1 mRNAs were measured by qRT-PCR and were normalized to the levels before treatment.

### Statistics

Statistical analyses were performed with Prism 8.0 (GraphPad Software, San Diego, CA, USA). Continuous variables were displayed as mean±standard deviation if normally distributed; otherwise, the medians and interquartile ranges were applied. Between-group comparisons were measured by Student’s *t* test or Mann–Whitney *U* test. For comparing between-group differences for mRNA stability at different time points, repeated measures analysis of variance was applied. All hypotheses were two-sided and all *P* values below 0.05 were deemed as statistical significance.

## Results

### Pulsatile ECMO alleviates myocardial inflammation and improves survival in rats with septic cardiomyopathy

**Figure 1:**
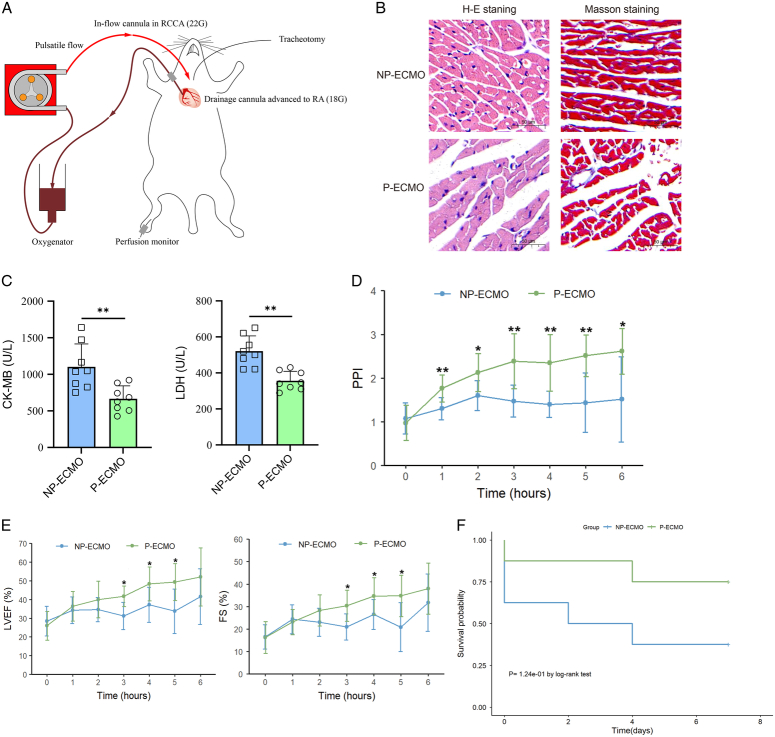
Pulsatile extracorporeal membrane oxygenation (ECMO) alleviates myocardial inflammation and improves survival in rats with septic cardiomyopathy. (A) The ECMO circuit in rats with septic cardiomyopathy. (B) Representative images of heart sections in septic cardiomyopathic rats after ECMO support with H&E (left) and Masson (right) staining were used. Scale bar represents 50 μm. (C) Serum CK-MB and LDH levels in rats of the two groups (*n*=8), **P<0.01. (D) Peripheral perfusion index (PPI) in rats supported with pulsatile or non-pulsatile ECMO within 6 h, *P<0.05, **P<0.01. (E) Comparison of LVEF (%) and FS (%) in rats at different time points during ECMO support, *P<0.05. (F) Survival analysis of rats supported by non-pulsatile or pulsatile ECMO. CK-MB, creatine kinase-myocardial band; FS, fraction shortening; H&E, hematoxylin and eosin; LDH, lactate dehydrogenase; LVEF, left ventricular ejectile fraction; NP-ECMO, non-pulsatile ECMO; P-ECMO, pulsatile ECMO; PPI, peripheral perfusion index.

To investigate the effects of pulsatility *in vivo*, we treated septic cardiomyopathic rats with pulsatile or non-pulsatile ECMO. We used a diagonal pump to generate pulsatility (Fig. 1A), and all ECMO circuits went smoothly without unsuccessful cannulation, cessation of treatment, or circulation failure. Pathological analyses showed that heart tissues in the pulsatile group presented lower inflammatory infiltration, attenuated myocardial injury as compared to the non-pulsatile group. Masson staining further revealed that damaged collagen was seen in the non-pulsatile group but not in the pulsatile group (Fig. 1B). By measuring the myocardial enzymes, we discovered that the CK-MB (creatine kinase-myocardial band) and lactate dehydrogenase (LDH) levels were significantly higher in the non-pulsatile group (Fig. 1C), while the effect was attenuated in rats exposed to pulsatility. To examine the peripheral tissue perfusion and cardiac function of the septic cardiomyopathic rats, ultrasonography was conducted. Our data showed that the pulsatile group rats had significantly higher PPI than did the non-pulsatile group (Fig. 1D), suggesting that pulsatility augments peripheral tissue perfusion in severe septic shock. Meanwhile, the LVEF levels and average FS were dramatically higher in pulsatile group rats during the course of ECMO support compared to the non-pulsatile group rats (Fig. 1E). Moreover, pulsatility was also associated with a longer overall survival time in septic cardiomyopathic rats (Fig. 1F).

### Pulsatile ECMO maintains the expression of ZO-1 in rats with septic cardiomyopathy

**Figure 2:**
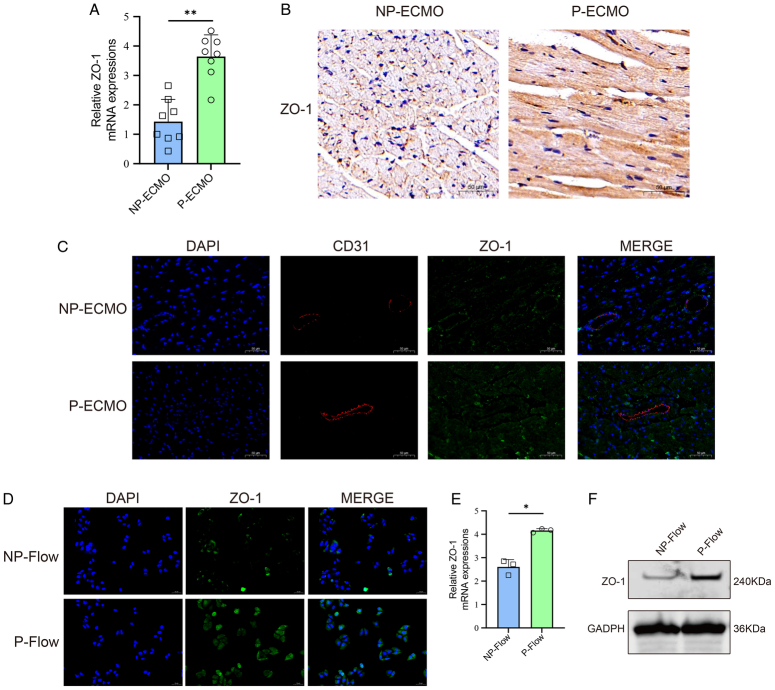
Pulsatile flow maintains the expression of ZO-1. (A) Relative expressions of ZO-1 in septic cardiomyopathic rats supported with pulsatile or non-pulsatile ECMO (*n*=8). (B) ZO-1 protein was measured by immunohistochemistry in heart tissues of septic cardiomyopathic rats after ECMO support. Scale bar represents 50 μm. (C) ZO-1 (green), CD31 (red), and DAPI (blue) staining by immunofluorescence in heart tissues showed that ECMO-generated pulsatile flow increased microvascular density and ZO-1 expression. Scale bar represents 50 μm. (D) ZO-1 (green) staining by immunofluorescence in CMECs indicated that the ZO-1 expression was up-regulated under pulsatile flow. (E) Quantitative reverse transcription-polymerase chain reaction (qRT-PCR) analysis of ZO-1 mRNA in CMECs, *P<0.05. (F) The expression of ZO-1 protein in CMECs was measured by Western blot. CMEC, cardiac microvascular endothelial cell; DAPI, 4′,6-diamidino2-phenylindole; ECMO, extracorporeal membrane oxygenation; GADPH, glyceraldehyde 3-phosphate dehydrogenase; mRNA, messenger RNA; NP, non-pulsatile; P, pulsatile.

To determine whether pulsatile flow maintains the endothelial structure in septic cardiomyopathy, the expression of ZO-1, an important marker of the endothelial structure, was measured. After ECMO support, expressions of ZO-1 of heart tissues in the pulsatile group were obviously higher as compared to the non-pulsatile group (Fig. 2A). Similar results were also seen in IHC analysis, revealing an increased expression of ZO-1 upon pulsatility (Fig. 2B). Interestingly, results from heart tissue immunofluorescence revealed that pulsatile ECMO support increased the density of microcirculatory capillary, which seems to be positively correlated with the ZO-1 expression (Fig. 2C). Further, we deduced that pulsatility affects the CMECs. *In vitro*, CMECs were exposed either to non-pulsatile flow or pulsatile flow. Immunofluorescence showed that the intercellular distribution of ZO-1 protein in non-pulsatile flow-treated CMECs was scattered and disrupted as compared to the pulsatile flow (Fig. 2D). This was further confirmed by qPCR and Western blot, which revealed lower expressions of ZO-1 in pulsatility-unexposed CMECs (Fig. 2E, F).

### Pulsatility promotes m^6^A modification but suppresses the PI3K–Akt signaling pathway in CMECs

**Figure 3:**
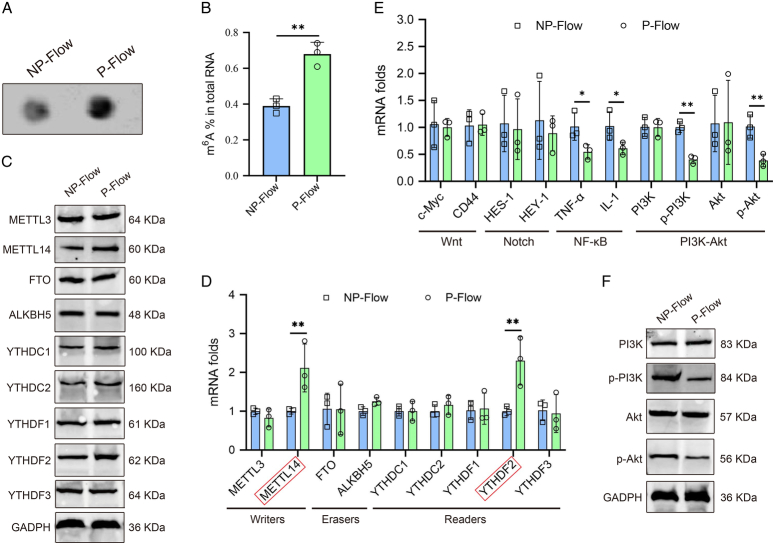
Pulsatile flow enhances m^6^A modification but inhibits the PI3K–Akt signaling pathway in CMECs. (A) Dot blot assay was carried out to examine the m^6^A levels of CMECs exposed to non-pulsatile or pulsatile flow. (B) Quantitative analysis of m^6^A levels of CMECs treated with non-pulsatile or pulsatile flow. (C) Western blot was performed to detect the expressions of different m^6^A writers, erasers, and readers in CMECs treated with non-pulsatile or pulsatile flow. (D) Messenger RNA (mRNA) expressions of various m^6^A writers, erasers, and readers in CMECs were measured using quantitative reverse transcription-polymerase chain reaction (qRT-PCR), **P<0.01. (E) qRT-PCR was conducted to screen out different pathway targets (Wnt, Notch, NF-κB, and PI3K–Akt signaling pathways in CMECs, *P<0.05, **P<0.01. (F) The expressions of PI3K–Akt signaling targets in CMECs were determined by Western blot. CMEC, cardiac microvascular endothelial cell; GADPH, glyceraldehyde 3-phosphate dehydrogenase; NP-Flow, non-pulsatile flow; P-Flow, pulsatile flow.

Dot blot analysis suggested that global m^6^A modification in CMECs treated with pulsatile flow was remarkably higher compared to those cells with non-pulsatile flow (Fig. 3A, B). Then we screened for the expressions of m^6^A writers, erasers, and readers, which were well-recognized in the m^6^A modification process upon flow patterns (non-pulsatile flow or pulsatile flow). Western blot and qPCR analyses revealed upregulation of METTL14 and YTHDF2 in pulsatility-managed CMECs (Fig. 3C, D), while the pulsatile flow did not markedly affect the expressions of m^6^A writers, erasers, and readers. We found that molecules associated with nuclear factor-kappa B (NF-κB) and PI3K–Akt signaling pathways were down-regulated in CMECs exposed to pulsatile flow (Fig. 3E). Results from the Western blotting confirmed the reduction of phosphorylation in PI3K and Akt (Fig. 3F), suggesting the inactivation of PI3K–Akt signaling pathway.

### Pulsatile flow facilitates METTL14-mediated m^6^A modification of ZO-1 and stabilizes the ZO-1 mRNA

**Figure 4:**
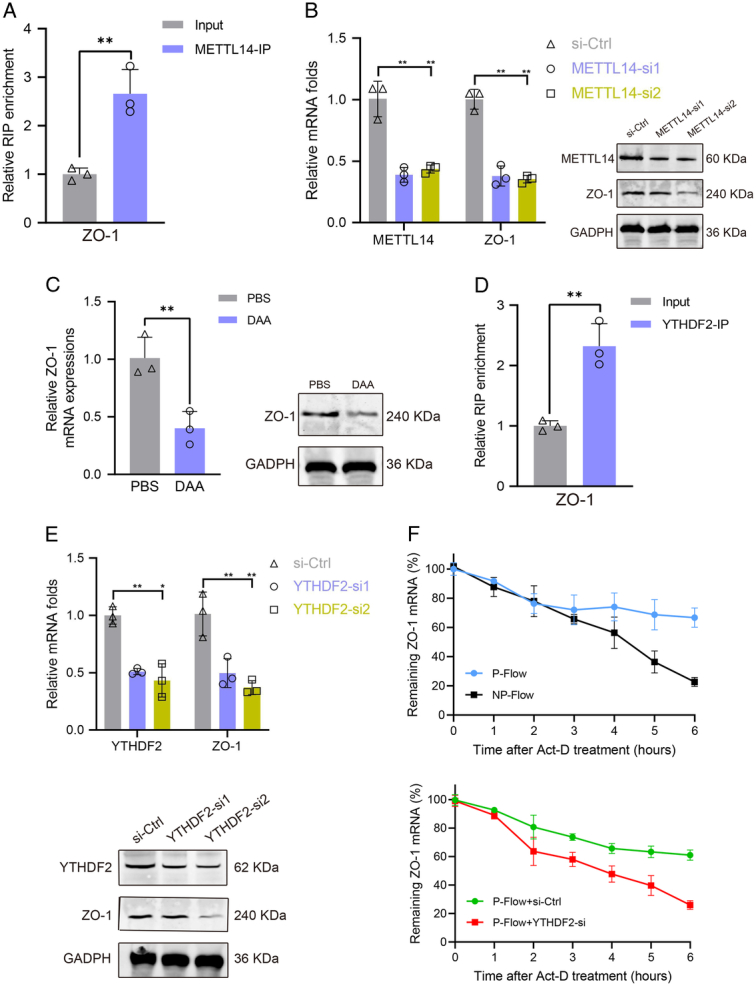
Pulsatile flow facilitates METTL14-mediated m^6^A modification of ZO-1 and stabilizes the ZO-1 messenger (mRNA). (A) RIP-qPCR assay of METTL14 interacting with ZO-1 mRNA in CMECs, **P<0.01. (B) CMECs that were treated with METTL14-siRNAs or control siRNAs were subject to quantitative reverse transcription-polymerase chain reaction (qRT-PCR) and Western blot detection of ZO-1 expression, **P<0.01. (C) CMECs were treated with 3-deazaadenosine (DAA), a global methylation inhibitor, and were then subject to qRT-PCR or Western blot measuring the expression of ZO-1, **P<0.01. (D) RIP-qPCR assay of YTHDF2 binding with ZO-1 mRNA in CMECs, **P<0.01. (E) CMECs that were treated with YTHDF2-siRNAs or control siRNAs were subject to qRT-PCR and Western blot analyses of the ZO-1 expression, **P<0.01. (F) CMECs were exposed to different conditions, and the remaining ZO-1 mRNAs were detected by qRT-PCR at different time points after actinomycin D treatment. Act-D, actinomycin D; CMEC, cardiac microvascular endothelial cell; Ctrl, control; DAA, 3-deazaadenosine; GADPH, glyceraldehyde 3-phosphate dehydrogenase; mRNA, messenger RNA; NP-Flow, non-pulsatile flow; P-Flow, pulsatile flow; PBS, phosphate-buffered saline; RIP, RNA immunoprecipitation; si, small interfering RNA.

To confirm that ZO-1 mRNA is the target of METTL14, RNA-RIP was performed, and the results reveal a direct interaction between METTL14 and ZO-1 mRNA (Fig. 4A). Silencing the expression of METTL14 obviously decreased the expressions of ZO-1 mRNA and protein in CMECs (Fig. 4B). Next, CMECs were cultivated with 3-deazaadenosine (DAA), a global methylation inhibitor, and the treatment down-regulated the expression of ZO-1 (Fig. 4C), suggesting that METTL14-mediated m^6^A modification positively regulates the expression of ZO-1.

RIP assays were conducted and the interaction between YTHDF2 and ZO-1 mRNA was observed (Fig. 4D). Knockdown of YTHDF2 significantly decreased the expression of ZO-1 in CMECs (Fig. 4E). Further, CMECs were treated with actinomycin D to block the process of transcription and results suggested that pulsatile flow extended the half-life of ZO-1 mRNA, while this effect of extension could be reversed by YTHDF2 silencing (Fig. 4F).

### Foxo1 is a transcription factor for the METTL14-mediated m^6^A modification regulated by the PI3K–Akt signaling pathway

**Figure 5:**
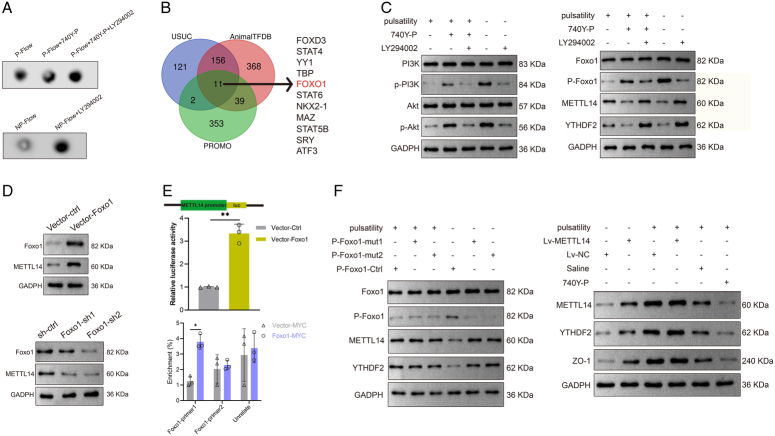
Foxo1 is a negative transcription factor for the METTL14 which is regulated by the PI3K–Akt signaling pathway. (A) Dot blot assay showed that the PI3K–Akt signaling agonist 740Y-P suppressed m^6^A modification in CMECs exposed to pulsatility, while this effect could be reversed by the inhibitor LY294002. Likewise, the m^6^A modification in CMECs could be induced by using the PI3K–Akt signaling inhibitor LY294002, as shown in the lower panel. (B) Venn diagram illustrating overlapping transcription factors predicted using three databases (USUC, AnimalTFDB, and PROMO). (C) The expressions of PI3K–Akt signaling targets, Foxo1, METTL14, and YTHDF2 in CMECs were measured using Western blot with indicated treatments. (D)The expression of METTL14 in CMECs was detected by Western blot upon Foxo1 over-expression and Foxo1 knockdown. (E) Dual-luciferase reporter and ChIP-qPCR assays to detect Foxo1 interacting with the binding regions of METTL14 promoter in CMECs. Anti-MYC-tag antibody was used to pull down the ectopic Foxo1, *P<0.05, **P<0.01. (I) The expressions of Foxo1, METTL14, YTHDF2, and ZO-1 were detected using Western blot under indicated treatments. ChIP, chromatin immunoprecipitation; CMEC, cardiac microvascular endothelial cell; lv, lentivirus; mut, mutation; NP-Flow, non-pulsatile flow; P-Flow, pulsatile flow; sh, short hairpin RNA; si, small interfering RNA.

740Y-P, the PI3K–Akt signaling agonist, abolished the effect of pulsatility for m^6^A modification in CMECs, while the effect was resurgent when the inhibitor LY294002 was added. Moreover, LY294002 induced m^6^A modification in CMECs exposed to non-pulsatile flow (Fig. 5A), indicating that PI3K–Akt signaling regulates the pulsatility-induced m^6^A modification. Next, we explored bioinformatics databases including USUC, AnimalTFDB, and PROMO, transcription factors that potentially bind the METTL14 promoter were then predicted, as shown in the Venn diagram (Fig. 5B). Since Foxo1 is the downstream molecule of PI3K–Akt signaling, we deduced that Foxo1 is a transcription factor for the METTL14-mediated m^6^A modification.

By using Western blotting, we confirmed that the PI3K–Akt signaling phosphorated Foxo1 (P-Foxo1), which down-regulated the expressions of METTL14 and YTHDF2; however, this effect could be reversed by adding LY294002. On the contrary, when the non-pulsatile flow-treated CMECs were co-cultured with LY294002, the expressions of METTL14 and YTHDF2 were up-regulated (Fig. 5C). Further, over-expression of Foxo1 significantly decreased the expression of METTL14, while silencing Foxo1 increased the METTL14 expression (Fig. 5D), implicating that Foxo1 is a negative regulator of METTL14.

To test whether Foxo1 is a direct transcription factor for METTL14, dual-luciferase reporter assays and chromatin immunoprecipitation (ChIP) were performed. The over-expression of Foxo1 promoted the METTL14-luciferase activity in CMECs, and the results of ChIP assay indicated that Foxo1 directly bound to the promoter of METTL14 in the region of Foxo1-primer that contained a putative binding element of METTL14 (Fig. 5E). Finally, we specifically used plasmids expressing mutant for phosphorylation (S250A or S313A) to dephosphorylate the expression of P-Foxo1, the expressions of METTL14 and YTHDF2 were regained. Meanwhile, the activation of PI3K–Akt signaling decreased the expression of downstream target ZO-1 protein *in vitro* (Fig. 5E).

### The m^6^A modification of ZO-1 reinforces ECMO treatment in septic cardiomyopathic rats

**Figure 6:**
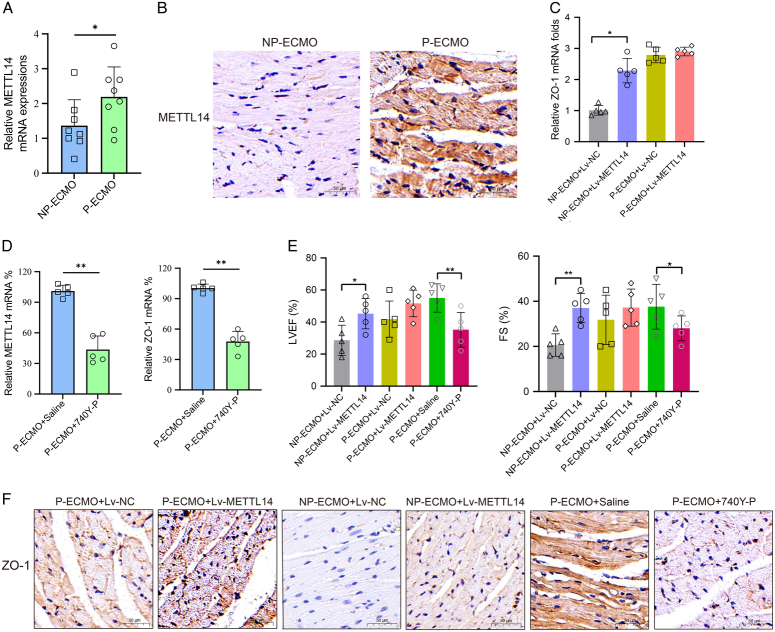
The METTL14-mediated m^6^A modification of ZO-1 reinforces ECMO treatment in septic cardiomyopathic rats. (A, B) Relative expression of METTL14 mRNA and protein in septic cardiomyopathic rats supported with pulsatile or non-pulsatile ECMO were determined using quantitative reverse transcription-polymerase chain reaction (qRT-PCR) (A) and immunohistochemistry (B), *P<0.05. Scale bar equals 50 μm. (C) The expression of ZO-1 mRNA upon METTL14 over-expression was detected using qRT-PCR with indicated treatments, *P<0.05. (D) Relative METTL14 mRNA and ZO-1 mRNA levels were measured with qRT-PCR in CMECs under pulsatile flow and PI3K–Akt signaling agonist 740Y-P, **P<0.01. (E) The LVEF and FS were measured using echocardiography in animal models with indicated treatments, *P<0.05, **P<0.01. (F) The expression of ZO-1 was detected with immunohistochemistry in heart tissues with different experimental settings. CMEC, cardiac microvascular endothelial cell; ECMO, extracorporeal membrane oxygenation; FS, fraction shortening; lv, lentivirus; LVEF, left ventricular ejection fraction; mRNA, messenger RNA; NC, normal control; NP, non-pulsatile; P, pulsatile.

After treating with pulsatile ECMO for 6 h, we observed elevated levels of METTL14 mRNA (Fig. 6A) and METTL14 protein (Fig. 6B) in the heart tissues. Then, by injecting lentiviral vector of METTL14 into septic cardiomyopathic rats which were treated with non-pulsatile flow, the expression of ZO-1 was significantly elevated (Fig. 6C). Further, 740Y-P (PI3K–Akt signaling agonist) was injected into those pulsatile ECMO-treated rats, the expressions of METTL14 and ZO-1(Fig. 6D) were markedly down-regulated, implicating that the PI3K–Akt signaling regulates the initiation of m^6^A methylation *in vivo*.

Next, gain-or-loss function experiments were performed. The LVEF and FS were remarkably improved in rats on non-pulsatile ECMO by over-expressing METTL14; meanwhile, the heart function was significantly damaged in pulsatile ECMO-treated rats by activating the PI3K–Akt signaling with 740Y-P (Fig. 6E). Similar results were also observed in immunohistological analyses. Injection of the lentiviral vector of METTL14 increased the expression of ZO-1 in rats undergoing non-pulsatile ECMO treatment, while injection of 740Y-P decreased the ZO-1 expression in rats on pulsatile ECMO (Fig. 6F).

## Discussion

**Figure 7:**
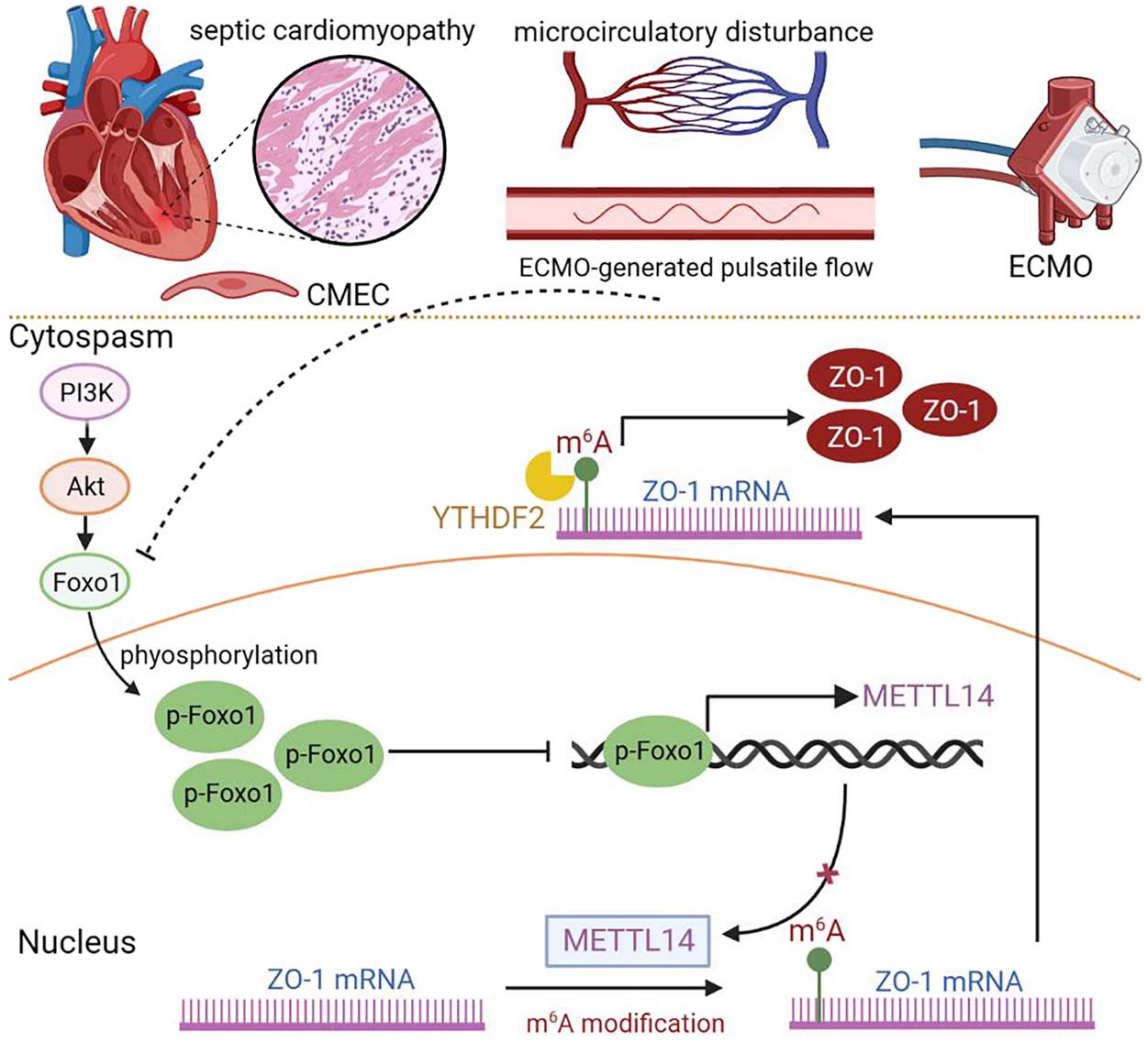
Schematic diagram of ECMO-generated pulsatile flow that increases the m^6^A modification of ZO-1 and alleviates septic cardiomyopathy. Notes: This figure was drawn with BioRender (http://www.biorender.com). CMEC, cardiac microvascular endothelial cells; ECMO, extracorporeal membrane oxygenation; mRNA, messenger RNA.

Emerging evidence has unraveled a close relationship between septic cardiomyopathy and m^6^A modification, which is relevant to sepsis-induced immune responses and subsequent cardiac injuries^14,21,22^. In this work, our findings reveal that the m^6^A modification of ZO-1 protects the endothelium and mitigates myocardial inflammation during septic cardiomyopathy. The ECMO-generated pulsatile flow significantly improves myocardial function and facilitates METTL14-mediated m^6^A modification. Mechanistically, METTL14-mediated m^6^A modification stabilizes ZO-1 mRNA dependent on the expression of YTHDF2, and Foxo1 is a transcription factor of METTL14. The pulsatile flow suppresses the PI3K–Akt signaling pathway, which initiates METTL14-mediated m^6^A modification, and Foxo1 binds to the promoter of METTL14, negatively regulating METTL14 transcription (Fig. 7).

Septic cardiomyopathy is one subtype of severe sepsis with a series of pathophysiological changes such as inflammation activation, myocardial dysfunction, metabolic disturbance, microcirculatory disturbance, and so forth^1,23^. As microcirculation is the final goal of sepsis resuscitation, recovery of microcirculatory perfusion is an ideal endpoint of treatment. Accumulating evidence has confirmed that peripheral malperfusion is associated with higher morbidity and mortality during shock^6,24,25^. Pathophysiologically, peripheral malperfusion is compromised by the activation of the sympathetic nervous system, increased vascular permeability, and inflammation cascade, which subsequently leads to visceral malperfusion, organ dysfunction, and finally death^26^. To overcome this dilemma, some researchers previously used diagonal pump-constructed ECMO circuit to provide pulsatile flow and improved survival in the canine model with cardiogenic shock^7,8^. The present work revealed that ECMO-generated pulsatile flow also reinforces tissue perfusion, improves cardiac function, and prolongs survival in rats with septic cardiomyopathy.

Septic cardiomyopathy also includes structural alterations in the endothelium, which is secondary to microcirculatory malperfusion, resulting in vascular permeability and microcirculatory disruption^27,28^. The significant role of ZO-1 on endothelial integrity, a representative molecule of the endothelial barrier, has been broadly acknowledged. Cheng *et al*.^29^ found that over-expression of ZO-1 attenuated intestinal barrier dysfunction and improved the survival rate of septic mice. Fan *et al*.^30^ reported that lactate deteriorated endothelial integrity through downregulation of ZO-1 in a heat shock protein A12 B-dependent way. In this study, we confirmed the protective effects of pulsatile flow on endothelial integrity in septic cardiomyopathic rats since the expression of ZO-1 and capillary density were largely increased.

METTL14, a pseudo-methyltransferase during m^6^A modification, diversely participates in the pathological process of cardiovascular diseases^31–33^. Nonetheless, upstream regulatory mechanisms of METTL14 were rarely acknowledged. Wang *et al*. reported that KAT2B stabilizes METTL14, which enhances m^6^A modification of Spi2a in macrophages and suppresses NF-κB pathway. The m^6^A modification of Spi2a inactivates macrophages and attenuates sepsis-induced myocardial injury in mice^33^. Our findings revealed Foxo1 as a transcription factor that binds to the METTL14 promoter, negatively decreasing the transcription of METTL14. Foxo1, recognized as a cancer-related transcription factor, is a forkhead box family member as well as one of the downstream molecules of the PI3K–Akt signaling^34^. Interestingly, we found that the PI3K–Akt signaling phosphorates Foxo1, thus reversed the METTL14-mediated m^6^A methylation. Meanwhile, our results suggest a flow-microcirculation communication in which the ECMO-induced pulsatile flow inactivates PI3K–Akt signaling in CMECs. However, how pulsatile flow suppresses the PI3K–Akt signaling is still unexplored and merits future investigations.

As broadly acknowledged, METTL4 has diverse biological functions, and we found that ECMO-provided pulsatile flow increases m^6^A levels in CMECs activated by METTL14. Under pulsatility, METTL14 behaves as a protective gene for septic cardiomyopathy by the m^6^A methylation of ZO-1, which stabilizes the ZO-1 mRNA upon the presence of YTHDF2. Though ZO-1 is recognized as the target of METTL14 under pulsatile flow, we could not confirm whether there are other target genes of METTL14 that affect the results. It is also noteworthy that silencing METTL14 globally was lethal in septic cardiomyopathic rats. METTL14 loss-of-function assays in septic cardiomyopathic rats were unsatisfactory. Other epitranscriptomic mechanisms may play a role, and more detailed mechanisms could be uncovered using multi-omics.

Although recent evidence showed the benefits of venoarterial (V-A) ECMO treatment in patients with septic cardiomyopathy, the survival rate was still disappointing^35,36^. One main drawback of conventional ECMO is the failure to provide biomimetic pulsatile flow which theoretically has little impact on microcirculatory resuscitation^37,38^. A recent development of VA-ECMO techniques is the modification of blood pump that enables pulsatile flow, such as the i-Cor and K-Beat system^39,40^. No consensus has ever been reached on their efficacy, and lack of standardization of pulsatile flow is the culprit^37^. Our work, however, first investigated the effects of pulsatile flow on animal models with septic cardiomyopathy and endothelial cells, with robust findings supporting the therapeutic effects of pulsatile flow on sepsis-induced microcirculatory failure. It would be preferable for the future development of mechanical circulatory support devices to include an option for pulsatility.

## Conclusion

The ECMO-generated pulsatile flow significantly improves myocardial function and induces the METTL14-mediated m^6^A modification to ZO-1 mRNA, which protects the endothelium and mitigates myocardial inflammation in septic cardiomyopathy. The pulsatile flow suppresses the PI3K–Akt signaling pathway, of which the downstream molecule Foxo1 suppresses the METTL14-mediated m^6^A modification. Therapeutic methods that enhance microcirculatory perfusion could be applied in future treatment for septic cardiomyopathy. Future mechanical circulatory support devices that include an option for pulsatility should be more optimal.

## Ethical approval

The experimental protocol was approved by the Institutional Animal Care Committee of the Guangdong Provincial People’s Hospital (reference no. KY-Z-2022-2553-01) in compliance with the Guide for the Care and Use of Laboratory Animals by the National Institute of Health (NIH) of the United States (revised in 1985).

## Consent

Not applicable.

## Sources of funding

This study was supported by grants from the Medical Scientific Research Foundation of Guangdong Province of China (Grant No. A2021433), the Traditional Chinese Medicine Bureau of Guangdong Province (Grant No. 20212071), City-College-Enterprise Joint Programs—Basic Research Plans in Dengfeng Hospitals of Guangzhou Science and Technology Plan Project (Grant No. SL2022A03J00253), Guangdong Basic and Applied Basic Research Foundation (Grant Nos. 2021A1515010280 and 2022A1515012170), and China Postdoctoral Science Foundation (Grant No. 2023M730743).

## Author contribution

S.Z.: data curation, investigation, formal analysis, and writing – original draft; K.W.: data curation and investigation; Z.Y. and Y.Q.: investigation and software; W.T.: data curation and methodology; Y.Z.: formal analysis, funding acquisition, and writing – original draft; S.A.S. and H.L.: investigation; J.Z.: investigation and formal analysis; K.Q.: conceptualization and resources; C.L.: resources and writing – reviewing and editing; G.L.: conceptualization, funding acquisition, writing – original draft, and writing – reviewing and editing, and supervision.

## Conflicts of interest disclosure

No author declares conflicts of interest.

## Research registration unique identifying number (UIN)

Not applicable.

## Guarantor

Guanhua Li, MD, PhD.

## Data availability statement

The data used to support the findings of this study are available from the corresponding author upon request at dr.liguanhua@hotmail.com

## Provenance and peer review

Not commissioned, externally peer-reviewed.

## Supplementary Material

SUPPLEMENTARY MATERIAL
