# Supplementary material for: Pulsatile flow increases METTL14-induced m6A modification and attenuates septic cardiomyopathy: an experimental study
Source: Int J Surg. 2024 Mar 27;110(7):4103–15. doi: 10.1097/JS9.0000000000001402 (PMC11254225; doi:10.1097/JS9.0000000000001402)
Supplement: SUPPLEMENTARY MATERIAL [file js9-110-4103-s002.doc]

| **Number** | **Gene name** | **Gene ID** | **Primers** | **Sequences** | **Length/bp** | **Tm/℃** | **Product(bp)** |
| --- | --- | --- | --- | --- | --- | --- | --- |
| 1 | ZO-1 | 292994 | ZO-1 Forward | GCTACCTTATTGAATGTCCCTGAT | 24 | 58.37 | 109 |
|  |  |  | ZO-1 Reverse | GAACTGAATGGTCTGATGCTAGTG | 24 | 59.43 |  |
| 2 | METTL3 | 361035 | METTL3 Forward | CACTTCAGACGGATTATCAACAAG | 24 | 57.9 | 113 |
|  |  |  | METTL3 Reverse | GCGTCAATTTCATAGTGGACATAC | 24 | 58.26 |  |
| 3 | METTL14 | 295428 | METTL14 Forward | GCACCTCGGTCATTTATATTTCTC | 24 | 57.77 | 146 |
|  |  |  | METTL14 Reverse | GTCTTTGTCTTTCCAGGATTGTTC | 24 | 58.24 |  |
| 4 | FTO | 291905 | FTO Forward | CAACAGGCACCTTGGATTATATCT | 24 | 58.62 | 137 |
|  |  |  | FTO Reverse | GTTGTGGATCTCTTCTCCTTGTTT | 24 | 58.93 |  |
| 5 | ALKBH5 | 303193 | ALKBH5 Forward | GATGAGATCACTCACTGCATACG | 23 | 59.02 | 85 |
|  |  |  | ALKBH5 Reverse | GTGCATCTAATCTTGTCTTCCTGA | 24 | 58.58 |  |
| 6 | YTHDC1 | 170956 | YTHDC1 Forward | AGAGTCAACCAGAAAAAACCTTGTC | 25 | 60.08 | 105 |
|  |  |  | YTHDC1 Reverse | GTCTATCAACTTCAAGCCCAATTC | 24 | 60.36 |  |
| 7 | YTHDC2 | 307446 | YTHDC2 Forward | TCTGGTAAGGTGAAAGAGAAATCC | 24 | 60.00 | 133 |
|  |  |  | YTHDC2 Reverse | GGAAACAAATTCCAGGTCTACATC | 24 | 60.11 |  |
| 8 | YTHDF1 | 296467 | YTHDF1 Forward | ACAGCCTAAGATGAAAACGAAGAG | 24 | 60.30 | 101 |
|  |  |  | YTHDF1 Reverse | CCTTGTTATCCCAAGTACCAATGT | 24 | 60.38 |  |
| 9 | YTHDF2 | 313053 | YTHDF2 Forward | GCACTGAAACTAGGTAGCACAGAA | 24 | 60.01 | 96 |
|  |  |  | YTHDF2 Reverse | AGCCACAATGTTACTAGTGATGGA | 24 | 59.95 |  |
| 10 | YTHDF3 | 361920 | YTHDF3 Forward | CAGATGGTGTTTTTAGTCAACCAG | 24 | 59.96 | 113 |
|  |  |  | YTHDF3 Reverse | ACTTGTTCCCCATGTAGAGAATC | 23 | 59.78 |  |
| 11 | c-Myc | 24577 | c-Myc Forward | GGAACTATGACCTCGACTACGACT | 24 | 60.08 | 129 |
|  |  |  | c-Myc Reverse | ATTTCTTCCAGATATCCTCACTGG | 24 | 59.87 |  |
| 12 | CD44 | 25406 | CD44 Forward | TGTACATCAGTCACAGACCTACCC | 24 | 60.34 | 108 |
|  |  |  | CD44 Reverse | GTGTGTTCTATACTCGCCCTTCTT | 24 | 60.08 |  |
| 13 | HES-1 | 29577 | HES-1 Forward | TCTGAGCACAGAAAGTCATCAAG | 23 | 60.95 | 118 |
|  |  |  | HES-1 Reverse | GGGGAGCTATCTTTCTTAAGTGCAT | 25 | 59.37 |  |
| 14 | HEY-1 | 155437 | HEY-1 Forward | GAGACCGAATCAATAACAGTTTGTC | 25 | 58.47 | 96 |
|  |  |  | HEY-1 Reverse | AGATCTCAGCTTTTTCTAGCTTCG | 24 | 58.89 |  |
| 15 | TNF-α | 24835 | TNF-α Forward | AGCAGATGGGCTGTACCTTATCTA | 24 | 60.51 | 121 |
|  |  |  | TNF-α Reverse | GCTGACTTTCTCCTGGTATGAAAT | 24 | 60.03 |  |
| 16 | IL-1 | 24494 | IL-1 Forward | AAGGGGAAGAATCTATACCTGTCC | 24 | 60.10 | 105 |
|  |  |  | IL-1 Reverse | CTTTTCCATCTTCTTCTTTGGGTA | 24 | 60.00 |  |
| 17 | PI3K | 298947 | PI3K Forward | TACCCTGGTGATTGAGAAGTGTAA | 24 | 59.93 | 100 |
|  |  |  | PI3K Reverse | GGTTTCATTGGATAGGACTGTAGG | 24 | 60.13 |  |
| 19 | Akt | 24185 | Akt Forward | GACCATGAACGAGTTTGAGTACCT | 24 | 60.8 | 112 |
|  |  |  | Akt Reverse | CTTGAGGATCTTCATGGCATAGTA | 24 | 59.65 |  |
| 20 | Foxo1-ChIP-1 | / | Foxo1-ChIP-1 Forward | CACCAACCAGCAAACCAAGATAAGT | 25 | 60 | 326 |
|  |  |  | Foxo1-ChIP-1 Reverse | GCCCCTTAGGATTGCTATCTGCA | 23 | 60.12 |  |

**Supplementary Table 1.** Primers for qRT-PCR.

| **Number** | **Targets** | **Gene ID** | **siRNAs** | **SS Sequence** | **AS Sequence** | **Length/bp** |
| --- | --- | --- | --- | --- | --- | --- |
| 1 | / | / | si-Control | UUCUCCGAACGUGUCACGUdTdT | ACGUGACACGUUCGGAGAAdTdT | 23 |
| 2 | METTL14 | 295428 | si-METTL14-1 | GGAUGAAGAUAAAGUAGAAGA | UUCUACUUUAUCUUCAUCCGU | 21 |
| 3 |  |  | si-METTL14-2 | GGACGAUAUCAUGAAGUUAGA | UAACUUCAUGAUAUCGUCCCA | 21 |
| 4 | YTHDF2 | 313053 | si-YTHDF2-1 | GGCUGAUAUUGCUAGCAAACC | UUUGCUAGCAAUAUCAGCCCA | 21 |
| 5 |  |  | si-YTHDF2-2 | CAGUGUUCCCAAAGUUGUAGG | UACAACUUUGGGAACACUGCU | 21 |
| 6 | / | / | sh-Control | CCTAAGGTTAAGTCGCCCTCG | | 21 |
| 7 | Foxo1 | 84482 | sh-Foxo1-1 | ATGGACAACAACAGTAAATTT | | 21 |
| 8 |  |  | sh-Foxo1-2 | CGCCAAACACCAGTCTAAATT | | 21 |

**Supplementary Table 2.** siRNA sequences.
